# Integrated Care Policies and Politics in Belgium: Conceptual, Contextual and Governance Linkages for More Effective Integrated Care Policy Management: Comment on "Integration or Fragmentation of Health Care? Examining Policies and Politics in a Belgian Case Study"

**DOI:** 10.34172/ijhpm.2023.7009

**Published:** 2023-07-30

**Authors:** Jean Macq

**Affiliations:** Institute of Health and Society (IRSS), UCLouvain, Brussels, Belgium

**Keywords:** Integrated Care, Policy Management, Governance, Place-Based, Multi-Level, Belgium

## Abstract

The study on the management of integrated care (IC) policies in Belgium from Martens et al illustrates the complex process of the political and stakeholder game in a country whose governance is changing as a result of successive state reforms. We argue that the way forward for putting health back at the centre of IC policy design and management is to improve three types of connections. First, the conceptual connections should help to articulate the different IC policies into a coherent overall picture. Second, contextual connections should allow for the adaptation of policies to different country contexts. This requires a new form of governance, ie, a place-based and adaptive form of governance. This can be developed, provided that a third connection, between the different levels of governance, is fully revised.

## Introduction

 Two years ago, Martens et al published in this journal the results of a study on integrated care (IC) policies management in Belgium.^1^ It illustrated the complex process of political and stakeholders gaming in a country where the governance of the state is becoming increasingly complicated as a result of repeated reforms.

 In such a context, IC policies design and implementation run the risk of being the result of political bargaining rather than a clear shared vision of how care should be organized. This is the case in Belgium, for example, when policies have to be designed to improve integration between primary care and mental health or with hospitals.

 At the same time, the jargon around IC can become instrumental for some stakeholders: “*integrated*” becomes, then, the buzzword to impose decisions that increase the power of certain professions or allow the hospital to develop outside its walls.

 Using the lenses of complex adaptive system to improve care integration within health (and social) care system has been advocated.^2,3^ This may be particularly relevant to overcome challenges such as those identified by Martens et al.^1^ In this approach, the policy-making and implementation process needs to adjust to the adaptive nature that results from the interactions between parts of the health (and social) care system. “muddling through,” “tinkering,” and “adaptation” become then key characteristics of policy-making and implementation process.

 Adaptation, to be accountable, needs transparency and informed debate. This is particularly challenging for IC policies management. Indeed, IC is a fuzzy concept and improvement of the integration of care, needs an action at the different levels of a health system (not only at operational, or inter-organizational level, but also at policy level).

 We argue that three type of connections are particularly challenging in improving IC in a context such as Belgium: a conceptual connection between policies, a contextual connection through strengthened place-based governance, and a multi-level connection between governance structures. This is coherent with recent commentaries and publications about IC in Belgium.^4-6^

## Conceptual Connections: From Integrated Care as a Fuzzy Concept to Connected Policies Within a Whole Picture

 In discourses and policies, IC is too often presented as a clear concept. However, when looking at content of policies, it appears fuzzy and subject to multiple interpretations.^7^ Its meaning differs, most often, in term of breath of ambitions and strategies to reach these ambitions. This is well illustrated in the article of Maerten et al.^1^ On the one hand, type 2 diabetus care trajectory have much narrower ambition with concrete financing mechanisms to achieve it. The primary care reform and more, the joint plan and pilot projects on IC have much broader ambition (quintuple aim). Both use the development of territorial governance as the frame to develop bottom-up strategies and to contribute to quintuple aim.

 The clarification of objectives and strategies in relation to specific policies is probably an initial step. This should allow for a coherent articulation of these policies. The IC policies analyzed by Martens et al makes it possible to illustrate different forms of connections.

 For some policies, desirable connections are more straightforward than others are. For example, type 2 diabetus care trajectory, because of the narrow breath of ambition can only be part of a “bigger picture.” Primary care reform and further, the joint plan could serve as “frame” to develop such policies, complementary to other (in mental health, etc). For that type of connection, there is a clear complementarity between pathways and territorial approaches to improve care integration.

 For other policies, the connection between primary care reform in Flanders and the joint plan on IC is less obvious. Indeed, both seek improved integration of care through territorial approach without clear agreement on the scale and mode of imbrication between territories defined in both policies. This form of incoherence has been recently identified as a key challenge in building IC.^8^

## Connection With the Context: Implementing “Place Based” Adaptive Form of Governance to Progressively Translate Policies Into Practice

 Even when conceptual connection between policies is achieved, traducing this from policy to action in a given context needs strong capacities at operational level. The most appropriate operational level to meet these capacities is probably the level of inter-organisational networks for a whole population living in a territory also called “integrated” place-base system of care.^9^ A key component to enable the best use of these skills is the development of new forms of governance at that level. Indeed, given the growing complexity of health systems, traditional approaches of governance do not suite realities anymore. The issue of responsiveness to various stakeholders (including population) becomes particularly important, when network or local system building is at stake. The challenge of responsiveness is particularly important to overcome barriers for integration such as “*lack of trust among key constituencies; insufficient understanding about changing environment and issues affecting healthcare organizations; emphasis on institutions, autonomy, independence; lack of system perspective; ambiguity about roles, responsibilities, relationships, accountabilities; readiness for change.*”^10^ The most appropriate form of governance to manage all those challenges is probably place-based governance.^9^ This form of governance groups stakeholders from different organisations for comprehensive solutions. It focusses on learning – by-doing to optimize policy translation to the local context.^11^ It is therefore adaptive by nature and build on principles that are proposed by Blanchet et al.^12^ First, an evaluation of the situation is coupled with the management of different forms of knowledge and information, ie, “hard” data (indicators of healthcare utilization and population needs) and “more practical and locally situated knowledge.”^9^ This enable the understanding of care provision by all stakeholders and the adaptation of strategies in context of uncertainties. Second, a strong leadership facilitates the negotiation between stakeholders through the management of their interdependency.^12^

 This mode of governance is probably a pre-requisite for the proper implementation of the three policies examined in Martens et al paper. Primary care zone (as part of primary care reform in Flanders) or IC pilot project zones (as part of joint plan) are the level to develop such a form of governance.

## Multi-level Connection Between Governance Structure: From Political Bargaining to the Support for the Translation of Policies Into Practice at Operational Level

 The development of place-based governance to manage “integrated” place-base system of care is far to be obvious. It depends of a coherent combination of decision, (governance) structures, skills and goodwill of many stakeholders.^1^ The recent covid crisis has shown that this was not the case in Belgium, a country were division of power has been the result of political bargaining rather than health-related reasoning. In such a situation, there is a clearly emerging agenda to connect better the different levels of state governance (namely the federal and federated levels). Indeed, distribution (with sometimes overlapping) of responsibilities in governance functions at multiple level such as local governments, regions, states and intergovernmental systems (such as European Union) give way to more diffused forms of governing. Reflecting and adapting the different level of governance to the others is the way forward for a federal country like Belgium. This should be conceived in such a way that it strengthens place-based governance to manage “integrated” place-base system and avoid the problems with the classical mismatch between policies and local realities whereby stakeholders including local authorities resist to it or translate it into practice.^13^

## Ethical issues

 Not applicable.

## Competing interests

 Author declares that he has no competing interests.
